# Personalisation - An Emergent Institutional Logic in Healthcare? Comment on "(Re) Making the Procrustean Bed? Standardization and Customization as Competing Logics in Healthcare"

**DOI:** 10.15171/ijhpm.2017.71

**Published:** 2017-06-20

**Authors:** Ewan Ferlie

**Affiliations:** School of Management and Business, King’s College London, London, UK.

**Keywords:** Institutional Logics (ILs), Standardisation, Personalization, Healthcare

## Abstract

This commentary on the recent think piece by Mannion and Exworthy reviews their core arguments, highlighting their suggestion that recent forces for personalization have emerged which may counterbalance the strong standardization wave which has been evident in many healthcare settings and systems over the last two decades. These forces for personalization can take very different forms. The commentary explores the authors’ suggestion that these themes can be fruitfully examined theoretically through an institutional logics (ILs) literature, which has recently been applied by some scholars to healthcare settings. This commentary outlines key premises of that theoretical tradition. Finally, the commentary makes suggestions for taking this IL influenced research agenda further, along with some issues to be addressed.

A recent ‘think piece’ by Mannion and Exworthy^1^ explores a balance or perhaps even tension that may be arising in current healthcare settings between what they term competing institutional logics (ILs) of standardization and customization in service delivery. So ILs here emerge as a key concept. Mannion and Exworthy adopt^1^ (p2) the definition of ILs as suggested by Thornton and Ocasio,^2^ namely: “the socially constructed, historical pattern of material practices, assumptions, values, beliefs, and rules by which individuals produce and reproduce their material subsistence, organize time and space, and provide meaning to their social reality” (some theoretical implications of using this definition will be explored later).

The Pro Crustean bed referred to in their title comes from Greek mythology and represents an arbitrary form of standardisation to which conformity is (in this case literally and brutally) forced. The image they present of a Pro Crustean bed being remade suggests that countervailing forces may be in play which could reduce – or at least rebalance – forces promoting high standardisation in health service delivery evident over the last 20 years.

Of course, their first argument that healthcare has been subjected to increased standardisation and a reduction in forms of traditional and tacit professional dominance which had been assumed to be strongly present up to the 1980s is by now a well trodden one. The UK healthcare sector – along with other health systems internationally – can indeed be characterised by the development of many explicit standards over the last twenty or so years. These developments include formal clinical guidelines, frameworks, protocols and checklists, all fuelled by the rise of the evidence-based medicine (EBM) movement. In addition, formal risk management and audit techniques have proliferated and can be seen theoretically as mundane and indirect but also pervasive technologies of organizational control.^3^ As seen theoretically from a Foucauldian framing (of which more later), such low level audit and risk management techniques record, categorise, ‘make knowable’ and also reshape day to day professional work practices. There has also been a growth of elaborate process controls in some major emergent fields, such as clinical governance and patient safety, which mix managerial with clinical elements.

An important novelty of the paper therefore lies in the authors’ counter argument that these forces of customization, personalisation and individualisation represent an emerging and opposite pole to the old standardization movement. Four different examples of these trends are given: (*i*) the rapid development of personalized medicine, including molecular markers, to target individual interventions; (*ii*) holistic forms of service delivery which try to treat each individual as a ‘whole person,’ including taking account of their values, wishes and lifestyle and reflecting this spirit in co-decision making; (*iii*) more market led notions of patient choice, and (*iv*) the taking of more responsibility by patients and publics for their own care (eg, personal health budgets; expert patient involvement especially within long term conditions).

The authors then argue that these novel trends – as one might expect - bring with them new issues and problems. There is, for example (p3), a rather brief discussion of a possible ‘responsibilization’ process whereby patients become – and are encouraged to become - more ‘calculating selves’ with a well developed self responsibility for their health (eg, eating well; taking exercise; employing stress reduction techniques). Here they cite the interesting work of Newman and Clarke^4^ exploring new forms of ‘publicness’ within contemporary public services. It is interesting to trace some core ideas in this domain in greater depth. Newman and Clarke’s^4^ Chapter 8 is entitled ‘Remaking Citizens: Transformation and Activation’ and draws out various different images of citizenship. ‘Responsible citizens’^4^ (pp164/165) take on high levels of responsibility for their own health and welfare, along with those of their family. They may (for example) build up savings or take out insurance policies, thereby reducing demands on public expenditure in the event of illness or old age. They may also be active citizens within voluntary organizations (some of which might well be health related) and help ‘govern the social’ in a broader way. A policy of co-production between health workers and users may reinforce these personalizing tendencies and discourses. Thus the recent introduction of Personalised Health Budgets in UK National Health Service (NHS) community health services is an excellent case in point where the user is now encouraged to co-construct with local health managers an individualised care package.

Behind these ideas lies some basic concepts drawn from Foucauldian social science. Space does not permit a full exposition of such a complex and multifaceted body of work here but two different subliteratures may be relevant. Mannion and Exworthy^1^ (p2)’s account of standardization cites the rise of audit and audit like techniques in public services organizations^3^ which can be seen as a mundane but ever more pervasive control technology. Audit reshapes day to day work practices and is consistent with one branch of Foucauldian analysis. Later Foucauldian work has also explored the contrasting and more subjective idea of the ‘technology of the self’ whereby individuals are seen as having the capacity to work on their own identities over a period of time and to transform them in a positive direction. So making ‘positive life style choices’ can clearly be seen in this light. The growth of therapeutic services and interventions may also be seen in this light. These questions are more fully explored in some interesting social science and broadly Foucauldian literature.^5^

Mannion and Exworthy^1^ (p3) note the extent of such responsibilization may be shaped by an individual’s background (and one might add their access to both financial and social capital), including their social and cultural background, beliefs and preferences. So the implication is that such capacity may develop highly unequally across different social groups.

What are the other theoretical as well as the health policy issues in their paper? How can these substantive trends be analysed in an intellectually engaging manner? Here their paper explicitly accesses the developing ILs literature stream within the discipline of organizational studies now recently applied to healthcare settings.^6,7^ Methodologically, this work tends to adopt qualitative and case study based designs, often of a comparative and longitudinal nature. It explores intermediate organizational processes more than cost structures or final clinical outcomes.

The IL literature is a recent substream of wider institutionalist literature. Institutionalism is a major school of organizational analysis which has long been used to examine long term and macro level processes of stability and also change across whole organizational fields. A classic study in healthcare is Scott colleagues’^8^ analysis of long term organizational change in the Bay area health system in the United states. While there is no space to present a full discussion of such a major and complex body of work, it can be noted that the school of institutionalism has developed strongly since the 1970s (classic early texts are Meyer and Rowan,^9^ and DiMaggio and Powell^10^). It takes a broadly Weberian approach in assuming the presence of relatively stable and rule bound large organizations.

The professions are seen as one important source of rules and social structure which may be highly influential across whole social fields,^2,8^ as is clearly the case in the healthcare sector given the powerful presence of institutional forms of medicine. In the United Kingdom healthcare system, for example, entry into and exit from the profession is controlled by the professionally dominated General Medical Council. Speciality specific professional associations (known as the Royal Colleges) play an important role in standard setting and education and training programmes nationally. There are also strong speciality specific international professional networks. Finally, the strong cognitive norms of bio science (as expressed in the conventions of peer review) also help shape the field intellectually.

An institutionalist perspective would often argue that market forces may be relatively weakly developed within particular fields with the result that there are not unambiguous competitive pressures on organizations to move to high levels of efficiency and maximise value creation for shareholders. Its perspective therefore differs from traditional models of competitive strategy coming from industrial economics.^11^ Rather, organizations may in such weakly developed or imperfect markets be more driven for a search for legitimacy than direct indicators of efficiency. High levels of legitimacy may in turn be signalled by imitating brand leaders or following the recipes of well-known management texts and gurus that circulate globally. Within UK healthcare, for example, senior managerial personnel within some organizations recently studied appear as open to the importing of texts and ideas from leading management consultants and leading American academics, often based in major Business Schools^12^ to complement traditional professional or public administration based forms of knowledge. Imitation within globalised management knowledge fields signals legitimacy and may be the sincerest form of flattery.^10^

Such processes of imitation would be likely to produce homogenous fields which are difficult to change. So the question arises: how do such organizational fields ever change? One view is that there may well be long standing periods of inertia or only highly incremental organizational change, but punctuated by occasional radical shifts.^13^ In such rare archetype transitions, some early work^13^ suggested formal structure, processes and underlying values would all shift and do so in a reinforcing manner within a relatively short period of time, then followed by another long stable period. Change to the underlying sphere of values and culture was particularly important but also difficult and complex. This archetype change proposition has clear similarities with the idea of paradigm change within scientific revolutions advanced within some sociology of science literature.^14^ Within healthcare studies influenced by institutionalism, one key theme has been exploring a possible archetype transition from professional to managerial dominance from the 1980s onwards (eg, for studies at field level, see Scott et al^8^ on the United States and Reay and Hinings^6^ on Alberta in Canada). Scholars are now thinking about different ways that multiple logics may relate to each other at the level of practice.^15^ Binder^16^ presents a case study of the different micro practices whereby practitioners in a supported housing organization responded to external funding pressures.

The commentary will now highlight four further interesting questions arising from the paper by Mannion and Exworthy.^1^ We have already referred to their definition of ILs (p3) which follows Thornton and Ocasio^2^ and is as follows: “the socially constructed, historical pattern of material practices, assumptions, values, beliefs, and rules by which individuals produce and reproduce their material subsistence, organize time and space, and provide meaning to their social reality.” Notice that the words ‘beliefs’ and ‘practices’ appear here as well as the more bureaucratic term ‘rules,’ and even here rules are seen as containing an element of social construction. This definition responds to criticisms of old institutionalist approaches that it had failed to consider the more subjective and practice related aspects of organizational life sufficiently.

Mannion and Exworthy^1^ (p3) call for the analytic focus to move down from the macro level which here refers to the whole healthcare field^6,8^ where much institutionalist analysis has historically taken place to consider further links with the meso (which is here defined as the level of the organization) and micro levels (here defined as the service delivery related level of the clinical team or individual clinician): ‘there remains an urgent need for more sustained work exploring how the competing logics of standardization and customization are articulated, adopted, blended and/or resisted on the front line of care delivery.’ Thus Currie and Spyriondis^7^ have considered how the individual enactment of novel hybrid (Nurse Consultant) roles is affected by competing managerial and professional macro logics which exist at the level of the wider field.

Thirdly, it is of note that the words ‘blended and resisted’ appear. The question which arises is: what is the nature of the relationship between different ILs? Is it one of antagonism or even contradiction? Or can tensions be managed through ‘work arounds’?^6^ Alternatively, is there a dialectical movement from one pole to another? Or can there be fruitful blending and hybridization?^17^ Some recent IL literature argues for the presence of multiple logics which somehow coexist^6,7^ rather than a pure transition between one dominant logic (professionalism) to another (managerialism). Front line health workers may seek (and also struggle) to reconcile financial/managerial logics coming from above with their own clinical vision within micro level practices of enactment (see Spitzmueller’s^18^ ethnography of the tension between a Medicaid logic of ‘fee for service’ and a clinical recovery model in an American mental health setting).

These questions are important as they suggest different scenarios for possible levels of conflict, coexistence or collaboration between professional and managerial logics in healthcare settings.

My fourth discussion point suggests a need for a more precise characterization of any emergent IL. ILs are generally seen as high level and associated with only a small number of major institutional fields,^19^ such as the family, the state, the market or indeed the professions.^20^ So ILs cannot emerge everywhere but need to be grounded in a strong social base. What is the major institutional field which is driving the personalization logic? Their four personalization forces specified are at present rather disparate (that is, science; co production and choice) but the growth of an influential market/informed consumer logic may be important and may be seen an alternative both to the traditional professionalism and new public management (NPM) style managerialist logics which are examined. A first challenge is therefore to specify the core of any such rising IL within healthcare in greater detail.

A final question arises: what are the implications of this framing for the conventional IL debate which has so far been about alternative managerial and professional ILs in healthcare?^6,8^ They as yet seem rather unclear. A market orientated/informed user logic does not restore old style professional dominance but neither does it crudely promote managerialisation, as it rather cuts out the middle manager.

## Conclusion

So this exploratory article by Mannion and Exworthy^1^ usefully explores possible limits to a strong recent standardization wave evident in healthcare settings and suggests that countervailing forces for personalisation may be coming into play. This is an important alternative perspective which needs to be explored further. Theoretically, it should trigger more research into healthcare organizations as seen from an additive IL perspective with the potential to add more variety to the academic study of healthcare organizations.

However, some IL literature – while it is clearly interesting to explore further, as suggested – remains at an abstract level and can be dry in tone. So it will be challenging to operationalise it within more applied study of healthcare settings, although some recent good studies^6,8^ are cited here. Reflecting on their analysis, it would be helpful to specify the nature of a possibly rising and market orientated IL coming from a growing social base of informed and active users/customers which they point to briefly in more detail.

## Ethical issues

Not applicable.

## Competing interests

Author declares that he has no competing interests.

## Author’s contribution

EF is the single author of the paper.
